# Efficacy and Safety of Robotic Surgery With Sentinel Lymph Node Navigation in Obese Patients With Endometrial Cancer: A Retrospective Study

**DOI:** 10.7759/cureus.82341

**Published:** 2025-04-15

**Authors:** Nozomi Fufuzono, Shinichi Togami, Mika Fukuda, Hiroaki Kobayashi

**Affiliations:** 1 Department of Obstetrics and Gynecology, Faculty of Medicine, Kagoshima University, Kagoshima, JPN

**Keywords:** endometrial cancer, obesity, robotic surgery, sentinel lymph node, surgical outcomes

## Abstract

Objective: This study aimed to evaluate the feasibility and safety of robotic-assisted surgery combined with sentinel lymph node navigation surgery (SNNS) in obese patients with International Federation of Gynecology and Obstetrics stage IA endometrial cancer, comparing surgical and oncologic outcomes between obese and non-obese patients.

Methods: This retrospective study evaluated patients who underwent robotic-assisted hysterectomy, bilateral salpingo-oophorectomy, and SNNS at Kagoshima University Hospital between July 2018 and December 2024. The surgical outcomes, including operative time, console time, blood loss, and postoperative complications, of patients with a body mass index (BMI) of ≥ 30 kg/m² and ≥ 35 kg/m² were compared with those of their non-obese counterparts.

Results: Among the 294 patients evaluated, 39.5% had BMI ≥ 30 kg/m². Compared to non-obese patients, obese patients had significantly longer surgery start to roll-in time (P = 0.0026) and higher intraoperative blood loss (P = 0.018), whereas no significant differences were observed in operative time, console time, or postoperative complications. Patients with BMI ≥ 35 kg/m² had significantly longer operative time (P = 0.03) and surgery start to roll-in time (P = 0.0039) but showed no increased risk of postoperative complications. No cases required conversion to laparotomy.

Conclusions: Robotic surgery with SNNS has minimal impact on the surgical outcomes of obese patients with endometrial cancer. Although obesity influences preoperative setup time and intraoperative blood loss, overall surgical safety is maintained. Thus, robotic surgery with SNNS is feasible and safe for these patients.

## Introduction

Endometrial cancer is one of the most prevalent gynecologic malignancies in Japan, with increasing incidence owing to lifestyle westernization [1]. However, patients have a good prognosis, with a high five-year survival rate of approximately 90% for early endometrial cancer [1], making quality-of-life-oriented treatment strategies crucial. Consequently, minimally invasive surgery (MIS) has been increasingly adopted in clinical practice. One major challenge in performing MIS for endometrial cancer is patient obesity.

Multiple epidemiological studies have identified obesity as a significant risk factor for endometrial cancer [2-4]. Previous cohort studies have demonstrated that robotic surgery in obese women offers advantages over open and laparoscopic surgery; these include reduced operative time, lower blood loss, and a higher number of harvested lymph nodes [5-8]. Moreover, the long-term outcomes of robotic surgery in obese patients are comparable to those in non-obese patients [9-11].

In Japan, the 2023 survey by the Japanese Ministry of Health, Labour, and Welfare showed that 21.1% of Japanese women had obesity (body mass index [BMI] ≥ 25 kg/m²). However, obesity (BMI ≥ 30 kg/m²) is extremely rare in the population aged 18 years, with a prevalence rate of only approximately 0.4% [12]. Thus, data on the outcomes of robotic surgery in young obese patients are limited. Furthermore, studies evaluating the efficacy of robotic surgery combined with sentinel lymph node navigation surgery (SNNS) in obese patients are scarce. In Japan, SNNS is currently most commonly applied in patients with FIGO 2008 stage IA endometrial cancer. For patients with more advanced stages, systematic pelvic and para-aortic lymphadenectomy remains the standard approach. Therefore, we limited our analysis to stage IA cases, which ensures a more uniform population and enhances the clinical relevance of our findings.

Thus, this study aimed to evaluate the feasibility and safety of robotic surgery with SNNS in obese Japanese women with endometrial cancer, comparing outcomes between obese and non-obese patients.

## Materials and methods

This retrospective study was approved by the Institutional Review Board of Kagoshima University Hospital (approval no. 240153) and was conducted according to the tenets of the Declaration of Helsinki.

Patients who underwent robotic-assisted simple hysterectomy (SH), bilateral salpingo-oophorectomy (BSO), and SNNS at Kagoshima University Hospital in Japan between July 2018 and December 2024 were evaluated. SNNS was performed for all patients in accordance with the standardized protocol established at our institution. The inclusion criterion was a postoperative pathologic diagnosis of International Federation of Gynecology and Obstetrics (FIGO) 2008 stage IA endometrial cancer. Patients with stage IB disease, sentinel lymph node (SN) metastasis, preoperative chemotherapy, and concurrent malignancies were excluded. Data were collected from electronic medical records.

All robotic surgeries were performed following a standardized technique. SNs were identified using a hybrid method combining technetium-99m radioisotope (RI) and indocyanine green (ICG). RI (111 MBq) was injected into the cervix at four quadrants (12, 3, 6, and 9 o’clock) the day before surgery, followed by lymphoscintigraphy and single-photon emission computed tomography/CT imaging. Immediately before surgery, 1 mL of 10× diluted ICG was injected into the cervix, and SNs were intraoperatively identified using a gamma probe and near-infrared fluorescence imaging. Patients with negative SNs did not undergo systematic lymphadenectomy.

Patients were placed in a 27-degree Trendelenburg position, with five trocars placed in a straight horizontal line at intervals of 7-9 cm. No uterine manipulator was used, and bilateral salpingectomy was performed prior to hysterectomy to prevent peritoneal dissemination via the fallopian tubes. Two robotic systems, namely, the da Vinci® Xi (Intuitive Surgical, Inc., Sunnyvale, USA) and hinotori™ (Medicaroid Corporation, Kobe, Japan), were utilized with no differences in procedural techniques.

Obesity was categorized based on BMI, with cutoffs of 30 kg/m² and 35 kg/m² for comparative analysis. Categorical variables were compared using the chi-square test, whereas continuous variables were compared using the Wilcoxon rank-sum test. All statistical analyses were performed using JMP software (JMP Pro version 14.0, SAS Institute Inc., Cary, NC, USA). P < 0.05 was considered significant.

## Results

A total of 294 patients with FIGO stage IA endometrial cancer who underwent robotic-assisted SH, BSO, and SNNS were evaluated. Among them, 39.5% (116/294) had a BMI ≥ 30 kg/m². Table 1 presents a comparison of the patient characteristics between the BMI groups. Age, final histological diagnosis, robotic system used, hospital stay, and recurrence were not significantly different between the BMI < 30 kg/m² and BMI ≥ 30 kg/m² groups.

**Table 1:** Comparison of patient characteristics between the BMI groups (BMI < 30 kg/m2 and ≥ 30 kg/m2) (n=294) Data are presented as n (%) or the median [range]. Data are presented as median [range] for continuous variables and n (%) for categorical variables. P-values for continuous variables were calculated using the Wilcoxon rank-sum test with normal approximation, and those for categorical variables were calculated using the chi-square test. “Z” indicates the Wilcoxon test statistic based on the normal approximation, and “χ²” indicates the chi-square test statistic. A p-value < 0.05 was considered statistically significant. BMI: Body mass index

Characteristic	BMI < 30 kg/m^2^ (n = 178)	BMI ≥ 30 kg/m^2^ (n = 116)	Test Statistic	P-value
Age (years)	57 [32–86]	56 [28–86]	Z = -1.52	0.13
Final pathology			χ² = 1.31	0.52
Endometrioid	176 (98.8%)	116 (100%)		
Serous	1 (0.6%)	0		
Clear cell	1 (0.6%)	0		
Robotic type			χ² = 0.16	0.69
da Vinci® Xi	152 (85.4%)	101 (87%)		
hinotori™ Surgical Robot System	26 (14.6%)	15 (13%)		
Length of hospital stay (days)	6 [3–13]	5 [3–13]	Z = -0.76	0.45
Recurrence			χ² = 1.31	0.33
No	177 (99.4%)	114 (98.3%)		
Yes	1 (0.6%)	2 (1.7%)		

Similarly, there were no significant differences between the BMI < 35 kg/m² and BMI ≥ 35 kg/m² groups, except for age (P = 0.039) (Table 2).

**Table 2:** Comparison of patient characteristics between the BMI groups (BMI< 35 kg/m2 and ≥ 35 kg/m2) (n=294) Data are presented as n (%) or the median [range]. Data are presented as median [range] for continuous variables and n (%) for categorical variables. P-values for continuous variables were calculated using the Wilcoxon rank-sum test with normal approximation, and those for categorical variables were calculated using the chi-square test. “Z” indicates the Wilcoxon test statistic based on the normal approximation, and “χ²” indicates the chi-square test statistic. A P-value < 0.05 was considered statistically significant. BMI: Body mass index

Characteristic	BMI < 35 kg/m^2^ (n = 236)	BMI ≥ 35 kg/m^2^ (n = 58)	Test Statistic		P-value
Age (years)	57 [28–86]	55 [28–80]	Z = -2.07		0.039
Final pathology			χ² = 0.50		0.19
Endometrioid	234 (99.2%)	116 (100%)			
Serous	1 (0.4%)	0			
Clear cell	1 (0.4%)	0			
Robotic type			χ² = 1.71		0.19
da Vinci® Xi	200 (79%)	53 (21%)			
hinotori™ Surgical Robot System	36 (87.8%)	5 (12.2%)			
Length of hospital stay (days)	6 [3–13]	5 [3–13]	Z = 0.52		0.6
Recurrence			χ² = 0.35		0.55
No	234 (99.2%)	57 (98.3%)			
Yes	2 (0.8%)	1 (1.7%)			

Table 3 compares the surgical outcomes between the BMI groups. The BMI < 30 kg/m² and BMI ≥ 30 kg/m² groups showed significant differences with respect to the time from surgery start to roll-in (P = 0.0026) and intraoperative blood loss (P = 0.018). In contrast, there were no significant differences in operative time, console time, roll-in to console start time, or postoperative complications. No cases required conversion to laparotomy or had intraoperative complications.

**Table 3:** Comparison of surgical outcomes between the BMI groups (BMI < 30 kg/m2 and ≥ 30 kg/m2) Data are presented as n (%) or the median [range]. Data are presented as median [range] for continuous variables and n (%) for categorical variables. P-values for continuous variables were calculated using the Wilcoxon rank-sum test with normal approximation, and those for categorical variables were calculated using the chi-square test. “Z” indicates the Wilcoxon test statistic based on the normal approximation, and “χ²” indicates the chi-square test statistic. A P-value < 0.05 was considered statistically significant. BMI: Body mass index

Characteristic	BMI < 30 kg/m^2^ (n = 178)	BMI ≥ 30 kg/m^2^ (n = 116)	Test Statistic	P-value
Operative time (min)	196 [68–527]	203 [84–555]	Z =1.13	0.26
Cockpit/console time (min)	144 [54–418]	149 [53–453]	Z =1.02	0.31
Time from start of surgery to roll in (min)	16 [6–80]	18 [7–119]	Z =3.01	0.0026
Time from roll in to cockpit/console start (min)	9 [4–50]	9 [3–43]	Z =-0.22	0.82
Blood loss (mL)	15 [3–335]	20 [2–453]	Z =2.36	0.018
Conversion to open surgery				
No	178 (100%)	116 (100%)		
Yes	0	0		
Intraoperative complications				
No	178 (100%)	116 (100%)		
Yes	0	0		
Postoperative complications			χ² = 0.94	0.33
No	175 (98.3%)	112 (96.5%)		
Yes	3 (1.7%)	4 (3.5%)		

Similarly, operative time (P = 0.03) and roll-in to console start time (P = 0.0039) were significantly longer in the BMI ≥ 35 kg/m² group than in the BMI < 35 kg/m² group. However, no significant differences were observed in console time, postoperative complications, or the rate of conversion to laparotomy (Table 4).

**Table 4:** Comparison of surgical outcomes between the BMI groups (BMI< 35 kg/m2 and ≥ 35 kg/m2) Data are presented as n (%) or the median [range]. Data are presented as median [range] for continuous variables and n (%) for categorical variables. P-values for continuous variables were calculated using the Wilcoxon rank-sum test with normal approximation, and those for categorical variables were calculated using the chi-square test. “Z” indicates the Wilcoxon test statistic based on the normal approximation, and “χ²” indicates the chi-square test statistic. A P-value < 0.05 was considered statistically significant. BMI: Body mass index

Characteristic	BMI < 35 kg/m^2^ (n = 236)	BMI ≥ 35 kg/m^2^ (n = 58)	Test Statistic	P-value
Operative time (min)	194 [68–527]	210 [98–555]	Z =2.17	0.03
Cockpit/console time (min)	144 [54–418]	156 [60–453]	Z =1.69	0.09
Time from start of surgery to roll in (min)	16 [6–80]	20 [7–119]	Z =2.89	0.0039
Time from roll in to cockpit/console start (min)	9 [4–50]	10 [3–25]	Z =0.59	0.55
Blood loss (mL)	20 [3–335]	28 [2–453]	Z =1.94	0.052
Conversion to open surgery				
No	236 (100%)	58 (100%)		
Yes	0	0		
Intraoperative complications				
No	236 (100%)	58 (100%)		
Yes	0	0		
Postoperative complications			χ² = 2.42	0.12
No	232 (98.3%)	55 (94.8%)		
Yes	4 (1.7%)	3 (5.2%)		

## Discussion

This study evaluated the impact of obesity on robotic-assisted hysterectomy with SNNS for FIGO stage IA endometrial cancer. The results showed that compared with their non-obese counterparts, patients with BMI ≥ 30 kg/m² had significantly longer time from start of surgery to roll-in and greater intraoperative blood loss, but operative time or console time was not significantly different. Additionally, in patients with BMI ≥ 35 kg/m², operative time and roll-in to console start time were significantly longer, but console time remained unchanged.

Obesity is a well-established risk factor for endometrial cancer, with a reported risk ratio of 1.52 [13,14]. Approximately 65% of patients undergoing hysterectomy for endometrial cancer had BMI ≥ 30 kg/m² [15], similar to findings in robotic surgery cohorts where 57.7% of patients are obese [16]. In contrast, the current study found that 39.5% of Japanese patients had BMI ≥ 30 kg/m², indicating a high prevalence of obesity within its cohort. The time from the start of surgery to roll-in was significantly longer (P = 0.0026), and intraoperative blood loss was significantly higher (P = 0.018) for patients with a BMI ≥ 30 kg/m² than for their non-obese counterparts. However, no significant differences were observed for total operative time, cockpit/console time, or roll-in to cockpit/console start time. Additionally, both operative time (P = 0.03) and roll-in to cockpit/console start time (P = 0.0039) were significantly longer in patients with a BMI ≥ 35 kg/m² than in their non-obese counterparts.

King et al. [17] reported that in robotic surgery for endometrial cancer, blood loss did not significantly differ based on obesity severity, but operative time was significantly prolonged in patients with BMI ≥ 50 kg/m². Similarly, Kadoch et al. [16] found that blood loss was increased (20 mL vs. 30 mL), setup time was extended, and total operating room time was prolonged in patients with BMI ≥ 40 kg/m². Furthermore, Asanoma et al. [18], in an evaluation of obese Japanese women, found that the degree of obesity was associated with increased setup time and prolonged wound closure time. Although some studies [16] have reported significant differences in blood loss among obese patients, the absolute difference is only approximately 10 mL, which is clinically insignificant. A key advantage of robotic surgery is that obesity has minimal impact on console time. However, as the degree of obesity increases, extended setup time due to trocar placement and other preparatory procedures contributes to a longer overall operative time, which should be considered in surgical planning.

This study found no significant association between the degree of obesity and hospital stay or postoperative complications. No correlation was observed between obesity severity and hospital stay or postoperative morbidity, consistent with previous reports [16-18]. Additionally, no cases of conversion to laparotomy or intraoperative complications occurred. This aligns with prior findings indicating no significant relationship between obesity and surgical conversion rates or intraoperative complications. These results support the safety and feasibility of robotic surgery for obese patients with endometrial cancer. Lindfors et al. [8] analyzed the long-term oncologic outcomes of obese patients with endometrial cancer who underwent either robotic or open surgery and found comparable long-term survival outcomes between the two approaches. Although the current study did not assess survival rates, recurrence rates did not significantly differ across BMI groups, supporting previous findings.

In the present study, no significant impact of obesity on surgical outcomes was observed between the two robotic platforms, da Vinci® Xi and hinotori™. Our previous study also demonstrated that there were no significant differences in operative time, estimated blood loss, or other perioperative factors between these two systems in surgeries for low-risk endometrial cancer [19].

A major strength of this study is its novel evaluation of the feasibility and safety of SNNS-assisted robotic surgery in obese Japanese women with endometrial cancer, an area with limited prior research. However, this study also has some limitations. First, it is a retrospective, single-center study, with data extracted from medical records, and this may have introduced selection bias. Second, the study focuses on short-term surgical outcomes, and long-term oncologic outcomes are not analyzed, requiring further investigation in future studies.

## Conclusions

In conclusion, among patients with FIGO stage IA endometrial cancer in Japan, 39.5% had BMI ≥ 30 kg/m². Importantly, the efficacy and safety of robotic surgery were not compromised in obese patients, thus confirming that robotic surgery with SNNS is a feasible and safe approach for these patients.
